# Dengue and Zika RNA-RNA interactomes reveal pro- and anti-viral RNA in human cells

**DOI:** 10.1186/s13059-023-03110-9

**Published:** 2023-12-05

**Authors:** Kuo-Chieh Liao, Xuping Xie, Anna Karin Beatrice Sundstrom, Xin Ni Lim, Kiat Kee Tan, Yu Zhang, Jing Zou, Amanda Makha Bifani, Hui Xian Poh, Jia Jia Chen, Wy Ching Ng, Su Ying Lim, Eng Eong Ooi, October M. Sessions, Yvonne Tay, Pei-Yong Shi, Roland G. Huber, Yue Wan

**Affiliations:** 1 Stem Cell and Regenerative Biology, Genome Institute of Singapore, Singapore, 138672 Singapore; 2 Department of Biochemistry and Molecular Biology, University of Texas Medical Branch, Galveston, TX 77555 USA; 3 Program in Emerging Infectious Diseases, Duke-NUS Graduate Medical School, 8 College Road, Singapore, 169857 Singapore; 4 Cancer Science Institute of Singapore, National University of Singapore, Singapore, 117599 Singapore; 5 Saw Swee Hock School of Public Health, National University of Singapore, Singapore, 117549 Singapore; 6 Department of Pharmacy, National University of Singapore, Singapore, 117559 Singapore; 7 Department of Biochemistry, Yong Loo Lin School of Medicine, National University of Singapore, Singapore, 117597 Singapore; 8 Biomolecular Function Discovery, Bioinformatics Institute (BII), Agency for Science, Technology and Research (A*STAR), Matrix #07-01, Singapore, 138671 Singapore; 9 School of Biological Sciences, Nanyang Technological University, Singapore, Singapore

## Abstract

**Background:**

Identifying host factors is key to understanding RNA virus pathogenicity. Besides proteins, RNAs can interact with virus genomes to impact replication.

**Results:**

Here, we use proximity ligation sequencing to identify virus-host RNA interactions for four strains of Zika virus (ZIKV) and one strain of dengue virus (DENV-1) in human cells. We find hundreds of coding and non-coding RNAs that bind to DENV and ZIKV viruses. Host RNAs tend to bind to single-stranded regions along the virus genomes according to hybridization energetics. Compared to SARS-CoV-2 interactors, ZIKV-interacting host RNAs tend to be downregulated upon virus infection. Knockdown of several short non-coding RNAs, including miR19a-3p, and 7SK RNA results in a decrease in viral replication, suggesting that they act as virus-permissive factors. In addition, the 3′UTR of DYNLT1 mRNA acts as a virus-restrictive factor by binding to the conserved dumbbell region on DENV and ZIKV 3′UTR to decrease virus replication. We also identify a conserved set of host RNAs that interacts with DENV, ZIKV, and SARS-CoV-2, suggesting that these RNAs are broadly important for RNA virus infection.

**Conclusions:**

This study demonstrates that host RNAs can impact virus replication in permissive and restrictive ways, expanding our understanding of host factors and RNA-based gene regulation during viral pathogenesis.

**Supplementary Information:**

The online version contains supplementary material available at 10.1186/s13059-023-03110-9.

## Backgrounds

Dengue (DENV) and Zika (ZIKV) viruses are important human pathogens belonging to the Flaviviridae family of RNA viruses. DENV is known to infect around 390 million people around the world annually [1], while ZIKV causes numerous diseases including microcephaly in infants [2]. Currently, limited treatments and vaccines are available and novel strategies and targets are urgently needed to develop therapeutics to treat these diseases. To achieve this, it is important to understand host factors and how they interact with DENV and ZIKV genomes during the viral life cycle.

DENV and ZIKV share a complex viral life cycle [3]. They initiate infection by binding to host cell receptors and undergoing endocytosis. Upon endosome acidification, the viral envelope fuses with the endosomal membrane, and the viral genome is released into the cytosol. This ~ 11 kb viral genome lacks poly-A tails and contains one open reading frame flanked by 5′ and 3′ untranslated regions (UTRs). Translation of viral genome produces a single polyprotein that is cleaved co- and post-translationally by cellular and viral proteases into 3 structural proteins (capsid, pre-membrane/membrane, and envelope) and 7 non-structural proteins (NS1, NS2A, NS2B, NS3, NS4A, NS4B, and NS5). These non-structural proteins are critical for the inhibition of host defense mechanisms and viral replication [4]. Newly synthesized viral RNAs can then be translated into additional viral proteins or packaged into progeny virions for subsequent rounds of infection.

DENV and ZIKV rely on host factors to establish successful infection and accomplish viral replication. Numerous siRNA and CRISPR screens have been performed in recent years to identify virus-restrictive and permissive factors [5–8]. In addition, host interactome studies reveal several important host factors for viral replication [9, 10]. However, most of the studies to date are focused on the role of host proteins. How DENV and ZIKV adapted the use of host RNAs for their own survival and how host cells use their RNAs to mount an antiviral response are still largely unknown. Additionally, whether a common set of host RNAs interact with different RNA viruses is unknown. Here, we used sequencing of psoralen crosslinked, ligated, and selected hybrids (SPLASH) to identify virus-host RNA interactions for 4 ZIKV and 1 DENV in human cells [11]. Functional characterization reveals diverse roles of these DENV and ZIKV interacting host RNAs in viral pathogenesis. For example, miR19a-3p associates with the ZIKV genome and promotes viral replication. Transfection of miR-19a-3p inhibitor resulted in a decrease in viral RNA abundance, suggesting that miR-19a-3p is a pro-viral factor. Anti-viral factors include DYNLT1 mRNA as its knockdown led to an increase in viral replication. We also compare DENV/ZIKV host interactors with SARS-CoV-2 interactors to find a common set of conserved RNA interactors. Altogether, this study highlights the functional significance of host RNAs in regulating host-virus interactions and viral pathogenesis.

## Results

### Virus genomes interact with hundreds of host RNAs

**Fig. 1:**
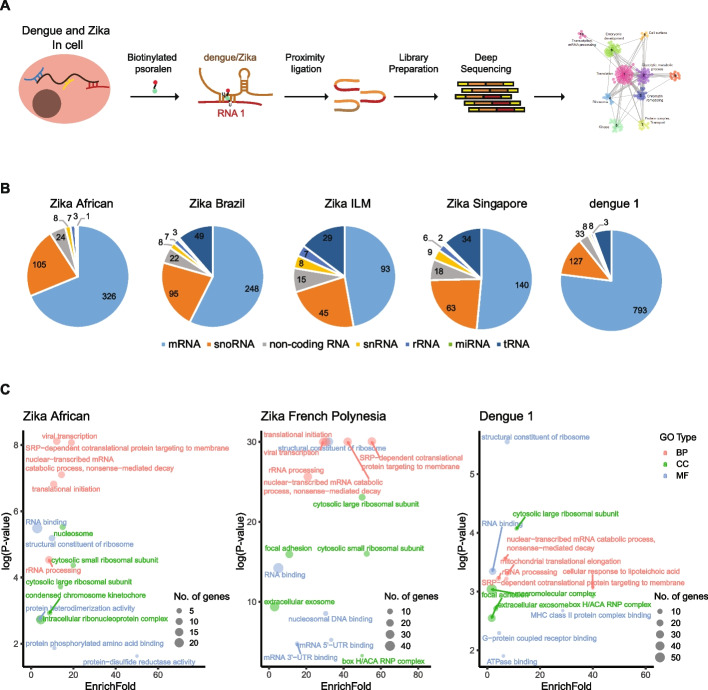
DENV and ZIKV genomes interact with hundreds of host RNAs in cells. **a** Experimental workflow to identify virus-host RNA interactions globally using biotinylated psoralen and proximity ligation sequencing. **b** Pie chart showing the number of RNAs from different RNA classes that interact with DENV and ZIKV viruses. **c** GO term enrichments of the host RNAs that interact with different strains of DENV and ZIKV viruses. The *Y*-axis indicates log (*p*-value) and the* X*-axis indicates the number of folds that the genes are enriched in binding to the virus genome as compared to the background

We have previously performed proximity ligation using biotinylated psoralen (SPLASH) in human neuronal progenitor cells cell (hNPCs) and Huh7 cells to study intramolecular RNA-RNA interactions within DENV and ZIKV genomes [12]. Besides capturing interactions within an RNA molecule, SPLASH also crosslinks intermolecular virus-host RNA interactions to provide information on the identity of host RNAs and the locations on which they bind to DENV and ZIKV RNA genomes. We performed analysis on four different strains of ZIKV in hNPCs and DENV-1 in Huh7 cells (Fig. 1a). The virus genomes show highly reproducible patterns of binding to host RNAs across biological replicates (Additional file 1: Fig. S1, *R* > 0.9). After filtering for highly consistent interactions across biological replicates, we observed that hundreds of human RNAs, including coding RNAs and long and short non-coding RNAs, interact with DENV and ZIKV genomes (Fig. 1b, Additional file 2: Table S1). These RNAs include Malat1, SND1, SSR2, SSR3, and SEC61A1, several of which were previously found in other siRNA and CRISPR host factor screens, confirming their importance in virus pathogenicity. Most of the viral interacting RNAs are localized in the cytosol (Additional file 1: Fig. S2a,b, Additional file 3: Table S2). GO term analysis of these factors showed that DENV and ZIKV binding RNAs are enriched for virus transcription and SRP signaling, agreeing with the importance of translation at the ER membrane for DENV and ZIKV replication [6] (Fig. 1c).

**Fig. 2:**
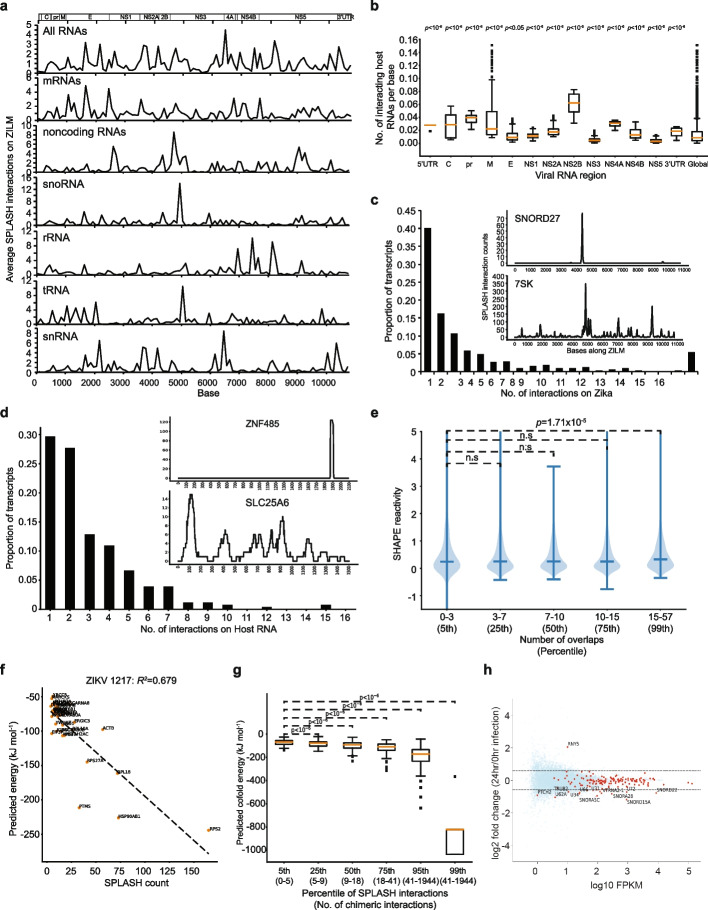
General properties of Zika-host RNA-RNA interactions. **a** Locations of interacting host RNAs on the viral genomes. Aggregate data and data classified by type of host RNA are shown. We observed differences in binding patterns by different classes of RNA along the genome. **b** The number of interactors in each coding region normalized by the length of the respective region. We observed the highest abundance of interactions in the NS2B-coding region. **c** Number of Zika genome binding sites for any specific host RNA (“Zika to host”). Most interactions bind at a specific, unique site. Promiscuous interactions of host RNA are present but considerably rarer. Inset, top: An example of a highly specific interaction between host RNA and virus genome, whereby SNORD27 only binds to a single region in ZIKV. Bottom: An example of promiscuous interaction between host RNA and virus genome whereby 7SK binds to many locations along ZIKV. **d** Number of interaction sites on host RNAs that bind to ZIKV genome (“host to Zika”). Data shows mostly unique interactions, but a higher propensity for 2 or more interaction sites. Inset, top: An example showing only 1 host region in ZNF485 interacting with the virus genome. Bottom: An example showing that many regions along SLC25A6 bind to the virus genome. **e** Sites along the Zika genome that show high numbers of interactions (99^th^ percentile of interactions) show a significant preference for single-stranded, high SHAPE reactivity segments inside virion particles. **f** Host RNAs interacting at ZIKV position 1217 with low predicted interaction energy corresponding to low observed read counts and high interaction energies coinciding with high observed read counts, indicating interaction energetics is a significant factor driving specific host-virus interactions. **g** Aggregate interaction energy statistics for percentiles of observed interaction counts, again with high interaction counts corresponding to high predicted interaction energy and significant differences between all classes. **h** Volcano plot showing the fold change in gene expression of host RNAs after 24 h of ZIKV infection. ZIKV interactors are colored as red dots, while the non-interactors are in blue. The dotted lines indicate a 1.5-fold change in gene expression

To better understand the nature of these virus-host interactions, we calculated the locations and frequency of virus-host interactions along with host and virus genomes. We observed that the entire genome of ZIKV interacts with host RNAs inside cells, although different classes of RNAs such as snoRNAs or mRNAs can bind to different regions along the virus genome (Fig. 2a). Across the different virus domains, we observed that the NS2B-coding region tends to be particularly enriched for binding to host RNAs (Fig. 2b). We observed that some of the virus-host RNA interactions are extremely specific, whereby only one region along the entire virus genome binds to the host RNA (Fig. 2c), or only one region along the host RNA binds to the virus genome (Fig. 2d). We also observed other RNAs that bind to the virus genome promiscuously by interacting with multiple regions along the viral genome (Fig. 2c), or by using multiple regions along the host RNA to interact with the genome (Fig. 2d). Along the host RNAs, we did not observe a preference for using either the 5′UTR, CDS, or 3′UTR to interact with the virus (Additional file 1: Fig. S2c), although bases that interact with ZIKV genomes are more evolutionarily conserved than non-interacting bases (Additional file 1: Fig. S2d). Additionally, abundant RNAs are also not significantly associated with high total interaction counts along the virus or high interaction counts at a specific site (Additional file 1: Fig. S2e), indicating that our observed virus-host interactions are not just a result of random interactions of the virus genome with abundant RNAs.

**Fig. 3:**
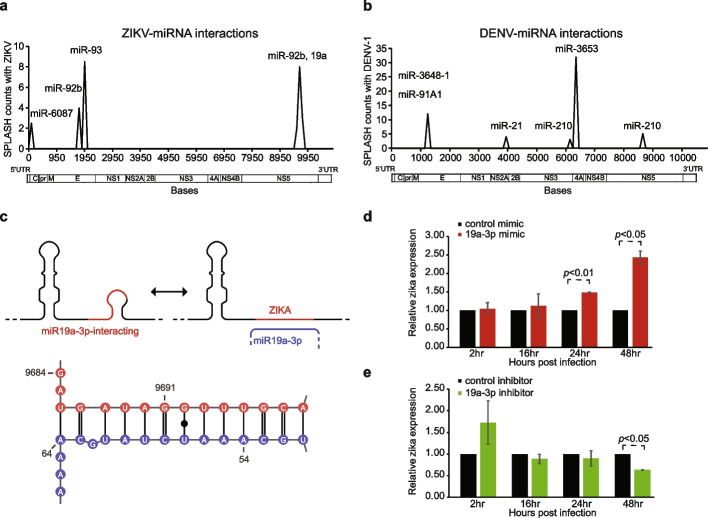
DENV and ZIKV interact with host miRNAs. **a**, **b** Line plot showing the locations along ZIKV (**a**) and DENV (**b**) that interact with host miRNAs. *Y*-axis indicates the number of interaction counts between the virus genome and a specific miRNA. The *X*-axis indicates the position along the virus genome. **c** Top, secondary structure modeling of ZIKV genome before and after miR-19a-3p binding. Bottom: Predicted ZIKV: miR-19a-3p interactions using the program RNAcofold. The ZIKV interacting sequence is in red and the miRNA sequence is in blue. **d**, **e** Bar charts showing the amount of Zika virus detected inside Huh7 cells using qPCR, 2, 16, 24, and 48 h post-infection in cells that are transfected with miR-19a-3p or control (**d**), or in cells that are transfected with miR-19a-3p inhibitor or control (**e**). The data in cells with over-expression of miR-19a-3p or inhibitor is normalized to its control

We observed that host RNAs frequently interact with initially single-stranded regions along the virus (Figs. 3c and 5b). As these regions could serve as intermolecular interaction hubs due to their relative structural accessibility, we tested whether this observation holds globally by determining the single-strand propensity of high versus low intermolecular interaction sites along ZIKV. We used SHAPE-MaP reactivities of ZIKV genome inside virion particles as it represents the structural state of the genome before being exposed to host RNAs inside cells [12, 13]. SHAPE-MaP reactivities show that regions that participate in high intermolecular interactions inside cells tend to be more single-stranded in virion particles (Fig. 2e), indicating that highly accessible regions along the genome could act as nucleating sites for host RNA interactions. We also observed that highly interacting ZIKV regions could bind to multiple host RNAs (Fig. 2f), suggesting that host RNAs could compete to interact with ZIKV and DENV genomes to result in different gene regulation outcomes. Interestingly, we observed that the strength of RNA binding to the virus genome (indicated by the number of SPLASH interactions) is correlated with predicted energetics of binding (Fig. 2g), and length of the interactions (Additional file 1: Fig. S2f), but not the GC content (Additional file 1: Fig. S2g). These analyses indicate that binding energetics is key to a host RNA’s ability to outcompete other RNAs when multiple RNAs can bind to the same site on the virus genome.

Previously, in our study of identifying SARS-CoV-2 interactome, we had observed that SARS-CoV-2-interacting RNAs are preferentially stabilized during infection even though many transcripts are downregulated [14]. To examine how the host RNA changes in gene expression upon ZIKV infection, we performed RNA sequencing experiments at 0 and 24 h post-ZIKV or upon mock infection in Huh7 cells. We observed that a similar number of host RNAs are up- (908) or down (911)-regulated by at least 1.5-fold in gene expression upon ZIKV infection (Fig. 2h). In contrast to SARS-CoV-2 interacting RNAs, ZIKV-interacting RNAs show a slight downregulation in gene expression upon ZIKV infection (Additional file 1: Fig. S2h). Out of 15 ZIKV-binding transcripts that show gene expression changes, 14 of them are downregulated, while only 1 (Y-RNA) RNA is upregulated (Additional file 4: Table S3, Fig. 2h). However, the functional mechanisms of how host RNAs are downregulated upon virus infection and how their downregulation impacts virus fitness remain to be studied.

### Virus genomes can interact with small non-coding RNAs to promote viral replication

In addition to mRNAs, we also observed that many short non-coding RNAs, including snoRNAs and miRNAs, interact with the viral genomes (Fig. 1b). We detected the binding of 5 miRNAs each to DENV and ZIKV genomes respectively (Fig. 3a, b). To determine whether any of our detected miRNAs could regulate virus pathogenicity, we focused on miR-19a-3p which was previously identified in an independent virus-host interaction study. Still, its impact on virus replication was unknown [15]. miR19a-3p is an important regulator in cancer, whereby it is known to target the key tumor suppressor PTEN [16]. Using the program RNA22 [17], we confirmed that miR19a-3p is predicted to hybridize strongly with the ZIKV genome at the base 9691, the same location where we detected miR-19a-3p:ZIKV interaction experimentally using SPLASH (Fig. 3c). To determine the impact of miR-19a-3p:ZIKV interaction on viral replication, we performed miRNA over-expression experiments by introducing a mimic of the miR19a-3p into Huh7 cells prior to ZIKV infection. We showed that the over-expression of miR-19a-3p mimic increased the relative amount of Zika RNA inside cells (Fig. 3d), suggesting that the miRNA acts as a virus-permissive factor. Additionally, the downregulation of miR-19a-3p in Huh7 cells by introducing an inhibitor before ZIKV infection resulted in a decrease in ZIKV genome abundance by about 50% over 48 h (Fig. 3e), confirming that miR-19a-3p promotes ZIKV replication.

To further investigate how miR-19a-3p can promote ZIKV growth, we studied the underlying RNA structures and alternative structures along the ZIKV genome in the absence of miR19a-3p binding. We observed two regions along the Zika genome can form alternative structures with upstream and downstream elements (Additional file 1: Fig. S3a), including a region (9860–9890 bases) that was previously identified to be highly structured and structurally conserved across different ZIKV (Additional file 1: Fig. S3a) [12]. As such, we hypothesize that miR-19a-3p binding to the ZIKV genome could potentially release this region to allow it to fold properly. Additionally, we also observed that miR-19a-3p levels do not significantly change upon ZIKV infection (Additional file 1: Fig. S3b). We hypothesized that the ZIKV genome could also serve as a sponge, whereby the binding of miR-19a-3p to ZIKV genome could result in having less miRNA being available to interact with its endogenous targets. Indeed, we observed a modest increase in PTEN mRNA levels, which is a target of miR-19a-3p, by an average of 20% in the presence of ZIKV (Additional file 1: Fig. S3c). Additionally, we also observed that the gene expression of other miR-19a-3p predicted targets is increased upon ZIKV infection (Additional file 1: Fig. S3d). These experiments indicate that miR-19a-3p could act as a pro-viral factor partially by rearranging ZIKV genome structure and by modulating the regulation of its downstream targets.

### Host RNAs that bind consistently across viruses are enriched for non-coding RNAs

**Fig. 4:**
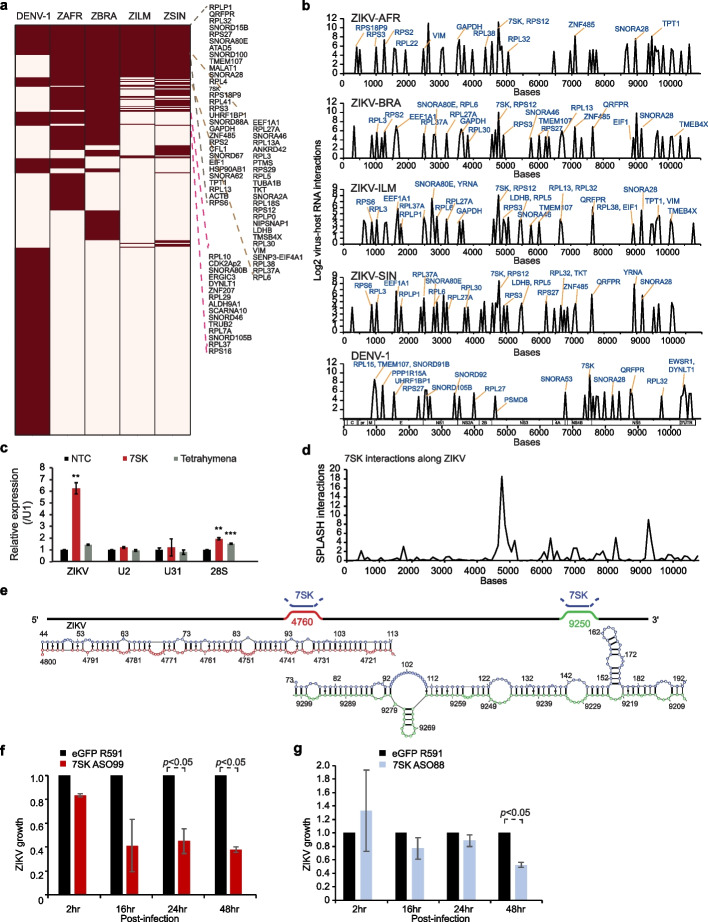
Noncoding RNAs interact with DENV and ZIKV genomes and impact virus fitness. **a** Heatmap showing the host RNAs that interact with DENV-1 and 4 strains of Zika. Host RNAs that interact with more than 3 viruses are listed on the right of the heatmap. **b** Locations of top 50 virus-host interaction sites along DENV-1 and the 4 ZIKV strains. Many of the top virus-host interaction sites are conserved across the viruses (shown in red). **c** qPCR analysis of ZIKV, U2, U31 and 28S rRNA pulled down by 7SK and tetrahymena RNA. Tetrahymena RNA is used as a negative control. **, *** indicate *p*-values ≤ 0.01 and 0.001 respectively, using Student’s *T*-test. **d** Locations along ZIKV that bind to 7SK RNA. The *Y*-axis indicates the number of SPLASH interactions between ZIKV and 7SK at that position. **e** Top, schematic of the strongest ZIKV-7SK interactions along the ZIKV genome. Bottom, predicted pairing interactions between ZIKV and 7SK using the program RNAcofold. 7SK sequences are shown in blue and the two ZIKV interaction sequences are in red and green respectively. **f**, **g** Bar charts showing the amount of ZIKV inside Huh7 cells at 2,16, 24, and 48 h post-infection, using qPCR analysis. ZIKV amount inside cells is decreased upon knockdown of 7SK using ASO99 (**f**) and after its interaction with 7SK is blocked using a 2’O-methylated anti-sense oligo to 7SK (ASO88) (**g**)

Highly abundant RNAs could interact with virus RNAs in a non-specific manner. As such, we identified RNAs that interact with viral RNAs with an interaction count that was higher than would be expected from their abundance (Additional file 1: Fig. S2e, Additional file 5: Table S4). Additionally, while specific host RNAs can bind to the different DENV and ZIKV viruses, we were curious to know whether there are host RNAs that can bind to at least 4 out of 5 viruses, and hence serve as general DENV/ZIKV interactors upon virus infection. We overlapped highly interacting RNAs that bind to the different viruses and identified 32 host RNAs that bind to all viruses, as well as 66 RNAs that bind to at least 4 out of 5 viruses (Fig. 4a, Additional file 1: Fig. S4a, Additional file 5: Table S4). Eleven out of 32 of the common interactors are noncoding RNAs, including snoRNAs. Interestingly, the location of the top binding peaks of these RNAs is highly conserved across the viruses (Fig. 4b), suggesting that their binding on the viral genomes is specific. To confirm that the virus-host interactions that we captured inside cells are indeed present, we performed pulldown experiments using biotinylated oligos against one of the non-coding RNAs that bind to multiple viruses (7SK). Pulldown and qPCR experiments showed that these host RNAs indeed interact with ZIKV as expected (Fig. 4c).

7SK is a member of the 7SK ribonucleoprotein (snRNP) complex that regulates polymerase II transcription and is also known to control the transcription of HIV [18, 19]. We observed extensive binding of the 5′end of 7SK along DENV and ZIKV genomes with the strongest interactions occurring within the NS3 and NS5 regions (bases 4760 and 9250, Fig. 4d, e). To determine whether 7SK RNA binding affects ZIKV replication, we designed two independent antisense oligos (ASOs) to knock down 7SK RNAs inside cells [20]. Transfection of ASOs against 7SK did not cause cellular toxicity (Additional file 1: Fig. S4b) and resulted in more than 90% knockdown of 7SK RNA for each ASO (Additional file 1: Fig. S4c,d). We observed that the downregulation of 7SK RNA resulted in a decrease in ZIKV levels inside cells (Fig. 4f, Additional file 1: Fig. S4e), suggesting that 7SK RNA promotes ZIKV replication. To confirm that it is indeed the interaction between 7SK-ZIKV that impacts ZIKV replication, we blocked 7SK and ZIKV interaction by using a 2’O-methylated antisense oligo that binds to 7SK. 2’O-methylated oligos have been used to block RNA-RNA interactions without triggering the RNase H cleavage mechanism [21]. We designed the 2’O-methylated oligo to target the strongest 7SK-ZIKV interactions in the cell and observed that blocking 7SK-ZIKV interactions resulted in a decrease in ZIKV expression without significantly impacting endogenous 7SK level (Fig. 4g, Additional file 1: Fig. S4f), consistent with the 7SK knockdown experiments. These experiments support our hypothesis that 7SK is a virus-permissive factor that facilitates ZIKV replication.

### The binding of DYNLT1 to DENV and ZIKV viruses inhibits viral replication

**Fig. 5:**
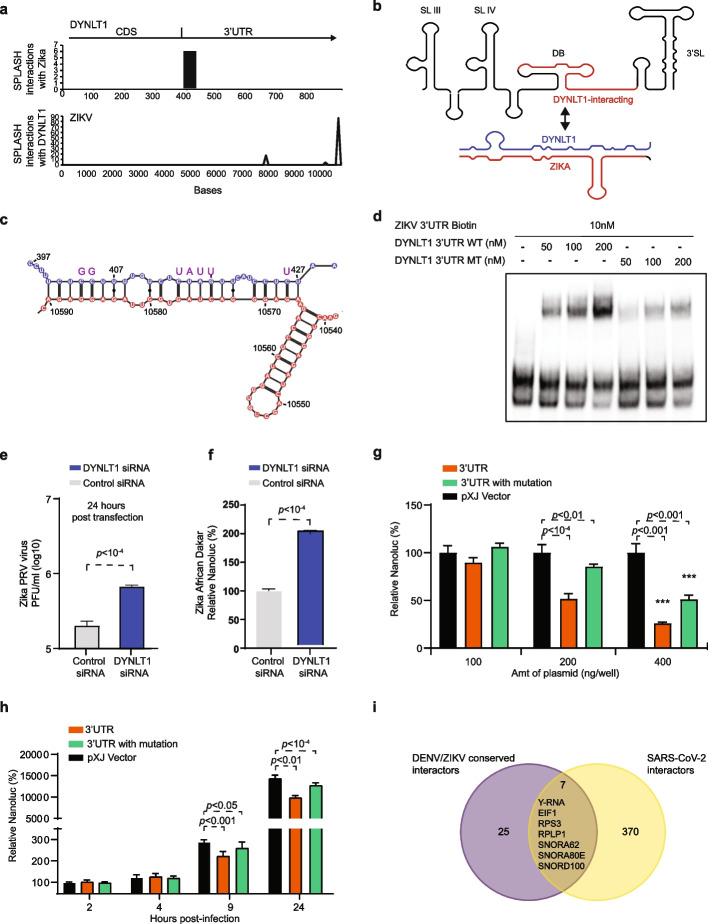
DYNLT1 is an anti-ZIKV factor that binds to ZIKV 3′UTRs. **a** Top, Location along DYNLT1 that binds to ZIKV. Bottom, Location along ZIKV that binds to DYNLT1. **b** Schematic and predicted RNA structures on ZIKV before and after DYNLT1 binding to the 3′UTR. The DYNLT1 sequence is in blue and the ZIKV sequence is in black and red. **c** Structure model of DYNLT1-ZIKV interaction using RNAcofold program. DYNLT1 RNA is in blue while the ZIKV genome is in red. The mutations on DYNLT1 3′UTR RNA are indicated in purple above the original bases. **d** EMSA data showing ZIKV and DYNLT1 RNA-RNA interactions using different amounts of WT and MT DYNLT1 3′UTR RNA. **e** Barplots showing the effect of DYNLT1 knockdown on ZIKV replication. Huh7 cells were transfected with 20 nM of DYNLT1 siRNA or a control siRNA. At 24 h post-transfection, cells were infected with recombinant ZIKV strain PRVABC59 at a multiplicity of infection (MOI) 0.5. The infectivities of the supernatants 1 day after infection were determined by plaque assay. Means and standard deviations (SDs) from six independent experiments are presented. **f** DYNLT1 knockdown reduces the replication of the ZIKV African lineage. Huh7 cells were transfected with 20 nM of DYNLT1 siRNA or a control siRNA. At 24 h post-transfection, cells were infected with a ZIKV strain Dakar containing a nanoluciferase gene. At 24 h post-infection, luciferase signals were measured. The relative luciferase signals were obtained by normalizing the luciferase readouts of the DYNLT1 siRNA-treated groups to those of the siRNA control. Means and SDs from five independent experiments are presented. **g** Overexpression of DYNLT1 3′UTR suppresses ZIKV replication. Huh7 cells were transfected with DYNLT 3′UTR, DYNLT1 3′UTR with mutation, or pXJ vector (100, 200, or 400 ng per well in a 24-well plate). At 24 h post-transfection, cells were infected with recombinant ZIKV strain PRVABC59 containing a nanoluciferase gene at an MOI of 0.5. At 24 h post-infection, luciferase signals were measured. The relative luciferase signals were obtained by normalizing the luciferase readouts of each group to those of the pXJ vector control. Means and SDs from four independent experiments are presented. **h** DYNLT1 3′UTR inhibits ZIKV replication. Huh7 cells were transfected with 200 ng of DYNLT 3′UTR, DYNLT1 3′UTR with mutation, or pXJ vector. At 24 h post-transfection, cells were infected with recombinant ZIKV strain PRVABC59 containing a nanoluciferase gene at an MOI of 0.5. At given time points, cells were harvested. The relative luciferase signals were obtained by normalizing the luciferase readouts of each group to those of control at 2 h post-infection. Means and SDs from four independent experiments are presented. **i** Venn diagram showing the amount of overlap between RNAs that interact with all 5 DENV and ZIKV viruses and RNAs that interact with SARS-CoV-2

In addition to non-coding RNAs, we also performed knockdown experiments of coding RNAs that are found to bind to DENV and ZIKV viruses, including RPL37A and EEF1A1 (Additional file 1: Fig. S5a). Knockdown of these RNAs resulted in a decrease in the amount of Zika being produced in the cells, indicating that they may promote viral replication (Additional file 1: Fig. S5b). Additionally, we observed that an mRNA coding for Dynein Light Chain Tctex-Type 1 (DYNLT1), which was previously associated with DENV infection, binds to ZAFR, ZBRA, and DENV-1 strains. SPLASH interaction reads indicated that the 3′UTR of the DYNLT1 binds to the dumbbell region in the viral 3′UTR of the viral genome (Fig. 5a, b). To validate this interaction, we performed an electrophoretic mobility shift assay (EMSA [22]) and showed that ZIKV was directly associated with DYNLT1 3′UTR (Fig. 5d). Furthermore, introducing mutations in DYNTL1 3′UTR reduced this physical interaction (Fig. 5c, d). To determine the biological significance of this interaction, we performed a knockdown of DYNLT1 using specific siRNAs and monitored virus replication upon lower levels of DYNLT1. RT-qPCR confirmed the DYNLT1 mRNA was reduced by > 60% from 24 to 72 h after siRNA transfection (Additional file 1: Fig. S5c) and that no cytotoxicity was detected upon DYNLT1 siRNA transfection (Additional file 1: Fig. S5d). Importantly, the replication of different ZIKV strains was significantly increased on Huh7 cells upon DYNLT1 knockdown (Fig. 5e, f). These data suggest that in contrast to miR19a-3p and 7SK, DYNLT1 may play a role in repressing viral replication.

As the SPLASH data showed that it is the 3′UTR of DYNLT1 that is primarily responsible for binding to ZIKV, we cloned the 3′UTR of DYNLT1 into a mammalian expression vector and monitored ZIKV replication upon the overexpression of the 3′UTR of DYNLT1 using the ZIKV strain PRVABC-59 containing a nanoluciferase gene (ZIKV PRV-Nluc). We observed that luciferase signals were inhibited by the DYNLT1 3′UTR in a dose-dependent manner 1 day post-infection (Fig. 5g). Additionally, the over-expression of DYNLT1 3′UTR resulted in a decrease in ZIKV amounts at early time points post-infection (9 h post-transfection, Fig. 5h), suggesting that DYNLT1 3′UTR may act on early stages of the virus life cycle. Interestingly, mutations that reduce ZIKV-DYNLT1 interactions inhibited ZIKV replication to a lesser extent (Fig. 5g), suggesting that the pairing between DYNLT1 and ZIKV is important. We also confirmed using RT-qPCR and Western blot analysis that overexpression of DYNLT1 3′UTR or 3′UTR with mutations did not alter the endogenous DYNLT1 mRNA and protein levels (Additional file 1: Fig. S5e,f). These results supported that DYNLT1 3′UTR suppresses ZIKV replication through the pairing between DYNLT1 3′UTR and viral 3′UTR. In addition to ZIKV 3′UTR, SPLASH results showed that DYNLT1 3′UTR can bind to DENV-1 3′UTR (Additional file 1: Fig. S5g). Additionally, overexpression of DYNLT1 3′UTR also inhibits the replication of both DENV-1 and DENV-2, while overexpression of mutant DYNLT1 3′UTR failed to suppress viral replication (Additional file 1: Fig. S5h,i), confirming our observation that DYNLT1 acts generally on both ZIKV and DENV viruses. However, how DYNLT1 binding suppresses ZIKV/DENV replication remains to be explored. By performing motif searches and filtering for RBPs with experimental binding evidence, we observed that 24 RBPs could bind near the DYNLT1 binding site (Additional file 6: Table S5). As such, more experiments are needed to confirm whether DYNLT1 3′UTR restricts virus growth by directly disrupting the dumbbell structure of its 3′UTR or by blocking one of the RBPs from binding to the virus.

Lastly, to determine whether the host RNAs that bind to DENV/ZIKV are unique to these flaviviruses or whether they can also interact with other RNA viruses such as SARS-CoV-2, we overlapped the conserved DENV/ZIKV interactors with SARS-CoV-2 interactors. Out of 32 RNAs that bind to DENV/ZIKV, we observed that 22% (7) of them, including Y-RNA, EIF1, RPS3, RPLP1, SNORA62, SNORA80E, SNORD100, bind to all three—DENV, ZIKV, and SARS-CoV-2—genomes (Fig. 5i). Some of these host factors, such as Y-RNA and RPLP1 are already known to be key pro-viral host factors involved in RIG-I activation and translation respectively, and our study suggests that there could be additional regulation at the level of RNA between the host and viral genomes.

## Discussion

When an RNA virus infects the cell, two events happen at the same time: the virus is trying to utilize host factors to maximize its replication, and in the meantime, the virus will encounter an anti-viral response from the host. As such, identifying host factors for virus pathogenicity is crucial in understanding the impact of RNA viruses on the host and how they can replicate. While most of the host factors identified to date are focused on proteins, the viral genomes also interact extensively with host RNAs through RNA base-pairing. Here we identify host RNAs that bind directly to ZIKV and DENV genomes to either restrict or enable the viruses to replicate. We showed that hundreds of mRNAs and noncoding RNAs can bind to the viruses and that the binding tends to occur on single-stranded regions of the virus and is energetics driven. While some host RNAs are virus-specific, others can bind universally across different DENV and ZIKV and may serve as pan-flavivirus host factors. We additionally showed examples of RNAs acting as both virus-permissive factors (miR-19a-3p, 7SK) and as virus-restrictive factors (DYNLT1). Although we have demonstrated the functional significance of these host RNAs, it remains to be determined whether these host RNAs have explicitly evolved to restrict viral replication, or they just happen to collaterally inhibit viral replication.

## Conclusions

By identifying functional RNA interactions, this work expands our understanding of host factors to include RNA as an important regulator of RNA virus replication.

## Methods

### Cells and viruses

Huh7 cells (RRID:CVCL_0336) were maintained in a high-glucose Dulbecco’s modified Eagle medium (DMEM) (Invitrogen, Carlsbad, CA) supplemented with 10% fetal bovine serum (FBS) (Hyclone Laboratories, Logan, UT), 1% penicillin–streptomycin (Invitrogen) at 37 °C with 5% CO_2_. The following infectious clone-derived viruses with or without the nanoluciferase (Nluc) reporter were used in this study: DENV-1 strain West Pac-Nluc, DENV-2 strain D2Y98P-Nluc, ZIKV strain Dakar-Nluc, ZIKV strain PRVABC59-Nluc, and ZIKV strain PRVABC59 [23, 24].

### siRNA transfection

10^5^ Huh7 cells were seeded into each well of a 24-well plate before transfection. On the next day, cells were transfected with DYNLT1 or control siRNA using the lipofectamine RNAiMAX Transfection Reagent according to the manufacturer’s instructions.

#### Electrophoretic Mobility Shift Assay (EMSA) [22]

Indicated amounts of DYNLT1 3′UTR RNA and biotinylated ZIKV ILM 3′UTR RNA are mixed in nuclease-free water, incubated at 90 °C for 1 min, put on ice immediately for 2 min, and incubated at room temperature for 5 min. Then, TMN buffer is added to reach 20 mM Tris–HCl (pH7.5), 100 mM NaCl, and 5 mM MgCl_2_ and this reaction is further incubated at 37 °C for 15 min. After incubation, these samples are resolved by native TBE PAGE and are transferred to Biodyne B nylon membrane. Subsequently, this blot is incubated with streptavidin–horseradish peroxidase (HRP) and is visualized with ChemiDoc Imaging systems (BioRad).

##### DYNLT1 3′UTR WT

CCTGCAGTCCAGCCTATGGCCTTTCTCCTTTTGTCTCTAGTTCATCCTCTAACCACCAGCCATGAATTCAGTGAACTCTTTTCTCATTCTCTTTGTTTTGTGGCACTTTCACAATGTAGAGGAAAAAACC

##### DYNLT1 3′UTR MUT

CCTGCAGTCCAGCCTATGGCCTTTCTGGTTTTGTCTTATTTTCATCCTTTAACCACCAGCCATGAATTCAGTGAACTCTTTTCTCATTCTCTTTGTTTTGTGGCACTTTCACAATGTAGAGGAAAAAACC

##### Biotinylated ZIKV ILM 3′UTR

GGAGATCAGCTGTGGATCTCCAGAAGAGGGACTAGTGGTTAGAGGAGACCCCCCGGAAAACGCAAAACAG

### DNA transfection

The 3′UTR and 3′UTR with mutations were cloned into a mammalian expression vector pXJ [25]. 8000 Huh7 cells were seeded into each well of a 24-well plate before transfection. On the next day, cells were transfected with plasmids that express either the wildtype or mutant 3′UTR using the X-tremeGENE™ 9 DNA Transfection Reagent (Roche) according to the manufacturer’s instructions.

#### Interactome mapping of viruses in human cells

Cells were infected with DENV and ZIKV viruses in 15-cm cell culture dishes. At indicated time points post-infection, these cells were washed twice in PBS and then incubated with 200 μM biotinylated psoralen and 0.01% digitonin in PBS for 10 min at 37 °C. These dishes were irradiated at 365 nm of UV for 20 min and total RNA was extracted from the cells using TRIzol reagent according to the manufacturer’s instructions. SPLASH libraries were prepared as described earlier [11].

### RT-qPCR

At given time points, cells were harvested and RNAs were extracted using the Qiagen RNeasy Mini Kit. The mRNA of DYNLT1 was quantified by RT-qPCR using the iTaq SYBR Green one-step kit (Bio-Rad) on the QuantStudio Real-Time PCR systems with primer pair DYNLT1_F (5′-ACACAGCAAAGTGAACCAG-3′) and DYNLT1_R (5′-GGTCAAATAGACAGTCCGAAG′3′). The mRNA levels of the housekeeping gene glyceraldehyde 3-phosphate dehydrogenase (GAPDH) determined by RT-qPCR with primer pair GAPDH-F (5′-CTCTGCTCCTCCTGTTCG) and GAPDH-R (5′-ACGACCAAATCCGTTGACTC-3′) were used as internal controls for cell number normalization.

### Reporter virus assay

At transfection, cells were infected with DENV or ZIKV reporter virus that contains Nluc. At given time points, cells were washed three times in PBS and lysed in 1 × cell culture lysis buffer. After all the samples were harvested, 50 μl cell lysates were transferred to a white opaque 96-well plate (Corning) are mixed with 50 μl luciferase substrates (Promega). For measuring the cell viability, 50 μl cell lysates were mixed with 50 μl CellTiter-Glo® 2.0 substrates (Promega). After 5-min incubation at room temperature, luciferase signals were measured using the Cytation 5 Cell Imaging Multi-Mode Reader (BioTek). Relative luciferase signals were normalized to the control at given time points.

### Plaque assay

At given time points, the culture medium from infected cells was clarified by centrifugation at 1000* g* for 5 min. Samples were stored at − 80 °C upon use. The number of infectious viruses was determined by standard cytopathogenic effect-based plaque assay on Vero cells [23]. Briefly, samples were 10-fold serially diluted in DMEM medium containing 2%FBS. 100 μl of the dilutions was added to a 24-well plate containing a monolayer of Vero cells. After 1 h of infection at 37 °C (plates were agitated gently every 15 min to ensure complete monolayer coverage during infection), 600 μl overlay medium containing 0.8% high viscosity carboxymethyl cellulose, 2% FBS, and 1% P/S was added to each well. The plates were incubated at 37 °C 5% CO_2_ for 4 days. Afterward, the cells were fixed in 3.7% formaldehyde solution at room temperature for 30 min before being stained with 1% crystal violet for 5 min. Visible plaques were counted and viral titers in PFU/ml were calculated.

### Computational analysis

Sequenced reads of 2 biological replicates were aligned against Zika genomes AY632535.2 (Uganda, MR766), KU497555.1 (Brazil, ZKV2015), KJ776791.2 (French Polynesia, H/PF/2013), KY241786.1 (Singapore, ZIKV-SG-116), DENV-1 genome EU081230.1 (Singapore, D1/SG/05K2402DK1/2005), and the human reference genome GRCh38.p12. Chimeric reads between the respective viruses and human RNAs were identified from this alignment using bwa-0.7.17 [26]. Subsequently, we identified the locations of host RNA interactions on viral genomes and the corresponding interaction sites on the host RNAs themselves. Analysis of replicates revealed consistent interaction counts (Additional file 1: Fig. S1) and was subsequently pooled for analysis.

RNAs were functionally classified according to the annotations in GRCh38.p12 (Fig. 1B).

Locations of interacting host RNAs on the viral genomes were aggregated and classified by type to reveal preferential host interacting locations along the Zika genomes (Fig. 2a). Subsequently, we analyzed the number of interactors in each coding region normalized by the length of the respective region (Fig. 2b). Analysis of the number of specific interaction sites per unique host-virus interaction was performed to determine whether specific RNAs interact in a specific or promiscuous manner. We determined the number of Zika genome binding sites for any specific host RNA (“Zika to host”), revealing that most interactions bind at a specific, unique site (Fig. 2c). To complement this analysis, we determined the number of interaction sites with Zika of any specific host RNA (“host to Zika”), which revealed mostly unique interactions but showed a higher propensity for 2 or more interaction sites (Fig. 2d).

In order to understand whether structural features of the viral genome determine the number of host interactions, we cross-referenced interaction sites with previously determined SHAPE reactivity data [12]. We find that for sites along the Zika genome that show high numbers of interactions (99^th^ percentile of interactions) a significant preference for single-stranded, high SHAPE reactivity segments is evident (Fig. 2e).

In order to assess whether observed interactions are plausibly driven by specific base-pair interactions, we analyzed the energetics of host-Zika RNA duplexes at the observed interaction sites using RNAcofold from the ViennaRNA software package to predict interaction energy [27]. Generally, a high correlation of predicted energetics with the number of observed interactions is revealed. Figure 2f shows host RNAs interacting at ZIKV position 1217 with low predicted interaction energy corresponding to low observed read counts and high interaction energies coinciding with high observed read counts as expected. Figure 2g shows aggregate interaction energy statistics for percentiles of observed interaction counts, again with high interaction counts corresponding to high predicted interaction energy and significant differences between classes. In addition to predicted energetics corresponding with observed read counts, we analyzed the relation of RNA transcript abundance (RPKM) with observed interaction sites. We identified no significant correlation between transcript abundance and the number of distinct virus genome interaction sites (SF 2b). The number of intermolecular base pairs and GC content of the interacting regions shows trends corresponding to the interaction energetics with an increased number of base pairs and increased GC content corresponding to higher observed read counts (SF 2c,d).

### Supplementary Information

**Additional file 1: Figure S1.** Quality metrics of SPLASH virus-host intermolecular interactions. **Figure S2.** Features of ZIKV- host RNA interactions. **Figure S3.** ZIKV:miRNA interaction statistics. **Figure S4.** ZIKV:non-coding RNA interaction statistics. **Figure S5.** DYNLT1 inhibits DENV and ZIKV replication.

**Additional file 2: Supplementary Table S1.** DENV ZIKV host interactions.

**Additional file 3: Supplementary Table S2.** Transcripts in different compartments.

**Additional file 4: Supplementary Table S3.** RNA sequencing ZIKV infection.

**Additional file 5: Supplementary Table S4.** Over-represented host interactors.

**Additional file 6: Supplementary Table S5.** RBP associated with ZIKV.

**Additional file 7.** Review history.

## Data Availability

Raw and processed data presented in this manuscript is available at GEO repository (GSE106483) [28]. The code is available at GitHub (https://github.com/rghuber/zika_splash/tree/zika_analysis_pub) [29] and Zenodo (10.5281/zenodo.10113321) [30].
