# Supplementary material for: Dengue and Zika RNA-RNA interactomes reveal pro- and anti-viral RNA in human cells
Source: Genome Biol. 2023 Dec 5;24:279. doi: 10.1186/s13059-023-03110-9 (PMC10696742; doi:10.1186/s13059-023-03110-9)
Supplement: Supplementary file 1 — Additional file 1: Figure S1. Quality metrics of SPLASH virus-host intermolecular interactions. Figure S2. Features of ZIKV- host RNA interactions. Figure S3. ZIKV:miRNA interaction statistics. Figure S4. ZIKV:non-coding RNA interaction statistics. Figure S5. DYNLT1 inhibits DENV and ZIKV replication. [file 13059_2023_3110_MOESM1_ESM.pdf]

Fig S1

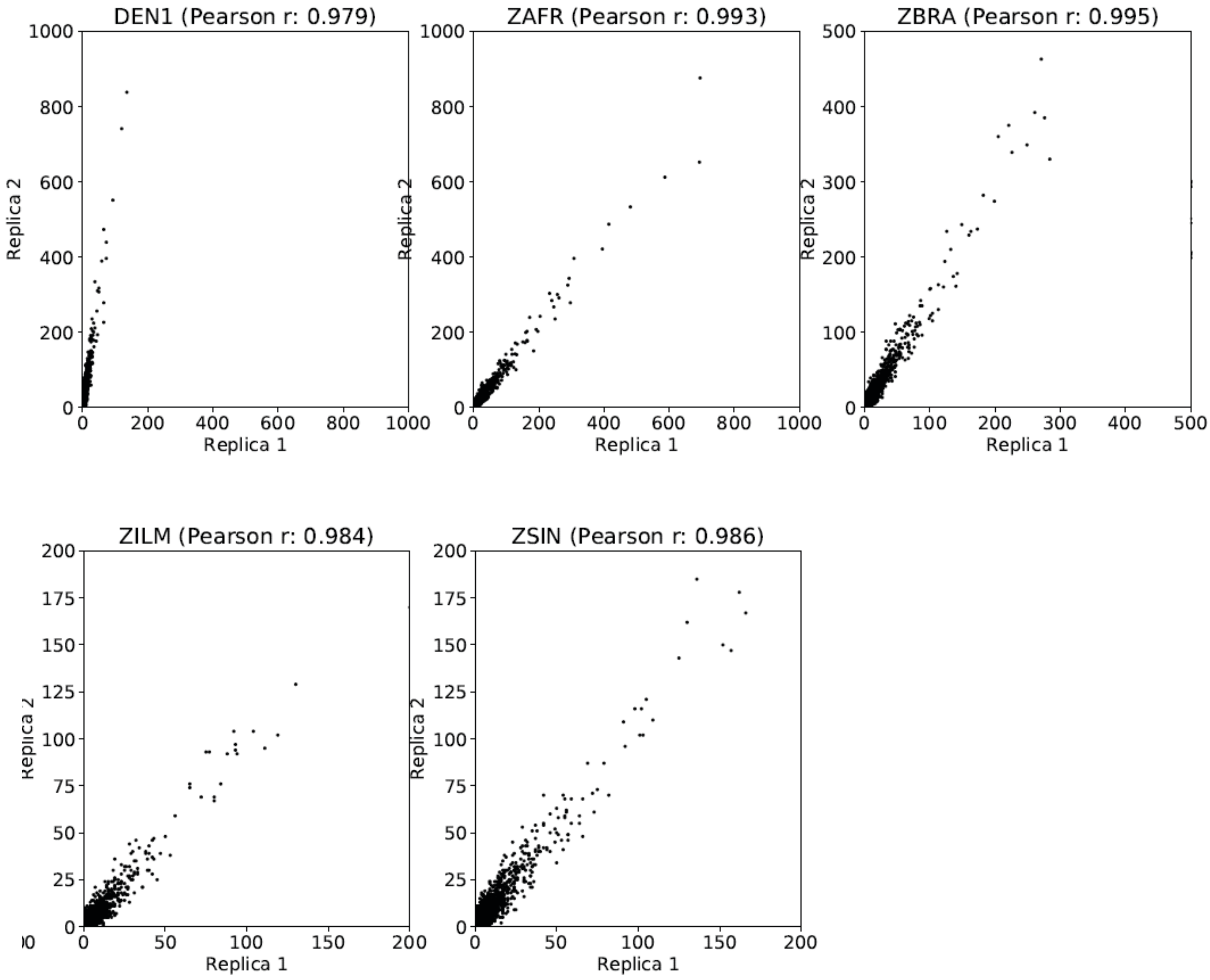
DEN1 (Pearson r: 0.979)
ZAFR (Pearson r: 0.993)
ZBRA (Pearson r: 0.995)
ZILM (Pearson r: 0.984)
ZSIN (Pearson r: 0.986)
Replica 1
Replica 2

Figure S1. Quality metrics of SPLASH virus-host intermolecular interactions. Analysis of biological replicates reveals consistent interaction counts between replicates for DENV and ZIKV. Hence, replicates were subsequently pooled for further analysis.

Fig S2

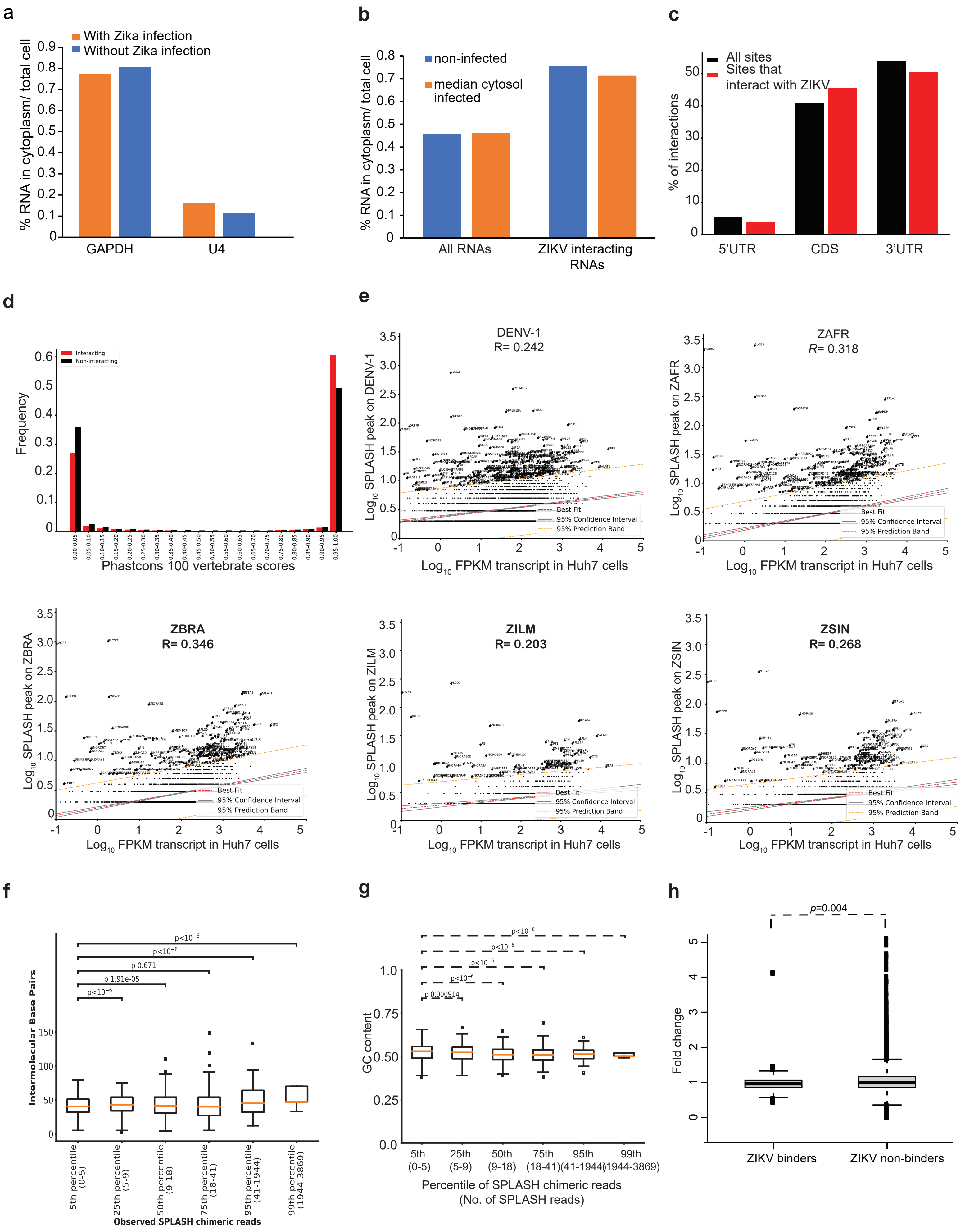
a
With Zika infection
Without Zika infection
% RNA in cytoplasm/ total cell
GAPDH
U4
b
non-infected
median cytosol infected
% RNA in cytoplasm/ total cell
All RNAs
ZIKV interacting RNAs
c
All sites
Sites that interact with ZIKV
% of interactions
5'UTR
CDS
3'UTR
d
Interacting
Non-interacting
Frequency
Phastcons 100 vertebrate scores
e
DENV-1
R= 0.242
ZAFR
R= 0.318
ZBRA
R= 0.346
ZILM
R= 0.203
ZSIN
R= 0.268
Log10 SPLASH peak on DENV-1
Log10 SPLASH peak on ZAFR
Log10 SPLASH peak on ZBRA
Log10 SPLASH peak on ZILM
Log10 SPLASH peak on ZSIN
Log10 FPKM transcript in Huh7 cells
Best Fit
95% Confidence Interval
95% Prediction Band
f
Intermolecular Base Pairs
p<10^-6
p 0.671
p 1.91e-05
5th percentile (0-5)
25th percentile (5-9)
50th percentile (9-18)
75th percentile (18-41)
95th percentile (41-1944)
99th percentile (1944-3869)
Observed SPLASH chimeric reads
g
GC content
p 0.000914
p<10^-6
5th (0-5)
25th (5-9)
50th (9-18)
75th (18-41)
95th (41-1944)
99th (1944-3869)
Percentile of SPLASH chimeric reads (No. of SPLASH reads)
h
p=0.004
Fold change
ZIKV binders
ZIKV non-binders

Figure S2. Features of ZIKV- host RNA interactions. **a**, Bar plots showing the percentage of GAPDH and U4 RNAs present in the cytosol versus in whole cell lysate, with (orange) and without (blue) Zika infection. **b**, Bar plots showing the percentage of RNA in cytosol versus whole cell lysate for all detected RNAs in the cell and for ZIKV interacting RNAs, with (orange) and without (blue) ZIKV infection. **c**, Analysis of interacting locations reveal no preference for 5' UTR, coding sequence, or 3' UTR regions. **d**, Histogram showing the distribution of Phastcons scores for 100 vertebrates for bases that are interacting versus non-interacting with ZIKV. **e**, Correlation of RNA transcript abundance (RPKM) with the number of virus interaction counts (SPLASH counts) for detected RNAs in the cell. We did not observe a strong correlation between transcript abundance and SPLASH interaction counts indicating that transcript abundance is not the main factor in driving virus-host RNA interactions. **f**, Boxplot showing the distribution of the number of paired bases between ZIKV and its interacting host RNAs at different quantiles of SPLASH interaction counts. The number of intermolecular base pairs of the interacting regions shows that highly interacting transcripts identified by SPLASH interact with each other with longer stretches of paired bases, as expected by energetics. **g**, GC content of the interacting regions is higher for strongly interacting transcripts. In agreement with observed energetics, increased GC content in the interaction region correlates with higher observed read counts. **h**, Boxplots showing the distribution of gene expression changes in host RNAs that bind (left) and does not bind (right) to the ZIKV genome, after the cells are infected with ZIKV for 24 hrs.

Fig S3

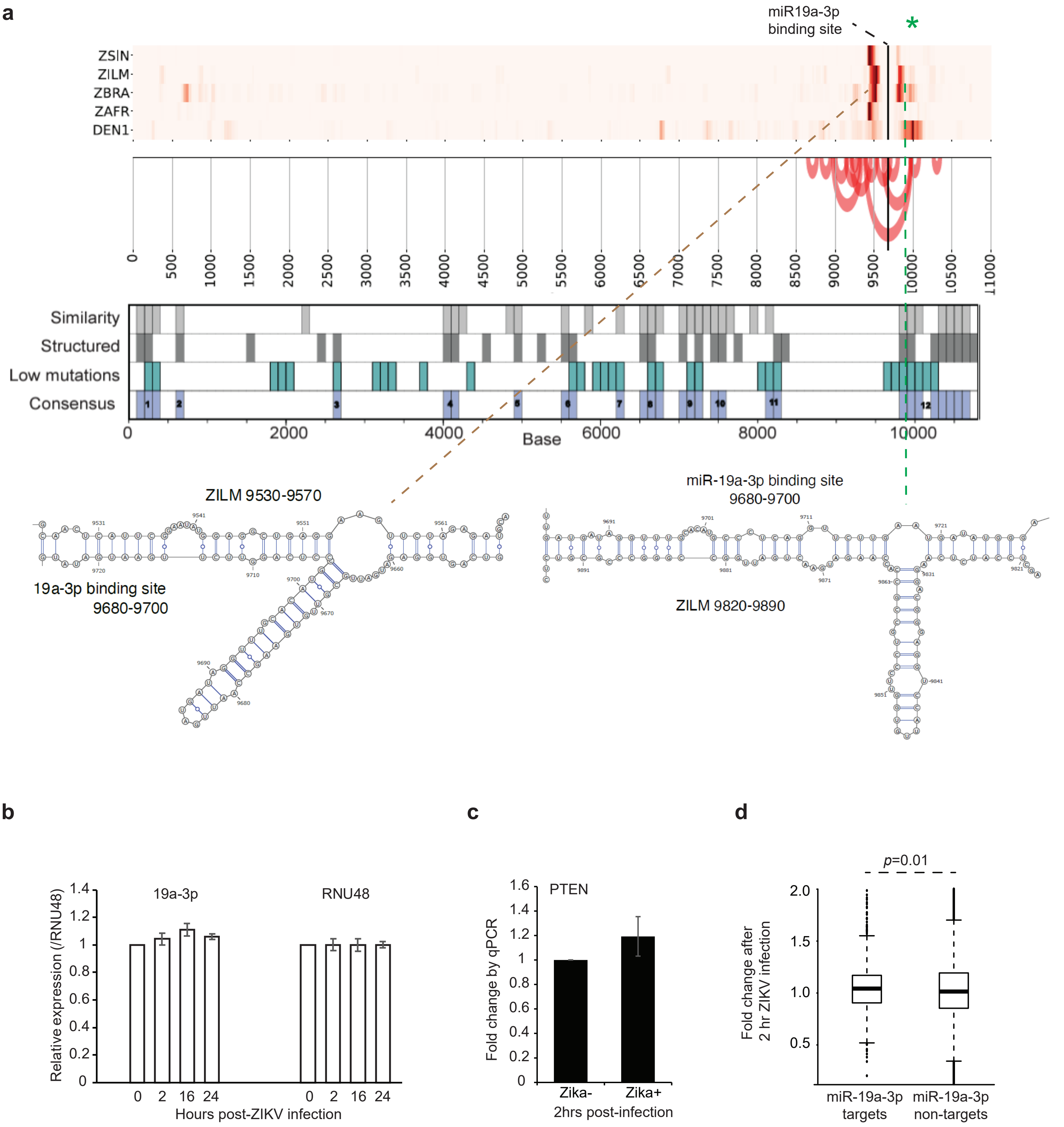

a
miR19a-3p
binding site
*
ZSIN
ZILM
ZBRA
ZAFR
DEN1
0
500
1000
1500
2000
2500
3000
3500
4000
4500
5000
5500
6000
6500
7000
7500
8000
8500
9000
9500
10000
10500
11000
Similarity
Structured
Low mutations
Consensus
1
2
3
4
5
6
7
8
9
10
11
12
0
2000
4000
6000
8000
10000
Base
ZILM 9530-9570
19a-3p binding site
9680-9700
miR-19a-3p binding site
9680-9700
ZILM 9820-9890
b
Relative expression (/RNU48)
1.4
1.2
1
0.8
0.6
0.4
0.2
0
19a-3p
RNU48
0 2 16 24
0 2 16 24
Hours post-ZIKV infection
c
Fold change by qPCR
1.6
1.4
1.2
1
0.8
0.6
0.4
0.2
0
PTEN
Zika-
Zika+
2hrs post-infection
d
p=0.01
Fold change after
2 hr ZIKV infection
2.0
1.5
1.0
0.5
miR-19a-3p
targets
miR-19a-3p
non-targets

Figure S3. ZIKV:miRNA interaction statistics. a, Top: Heatmap showing the bases along the ZIKV-ILM genome that interacts with the miR-19a-3p binding site. The black line represents the miR-19a-3p binding site. The green asterisk indicates the Zika bases that can interact with miR-19a-3p binding site and are in a highly structure conserved region. Upper Middle: Arc plots showing the RNA-RNA interactions along the ZIKV genome in this region. Lower Middle; Schematic showing the locations along ZIKV genomes that are structurally conserved (light grey), highly structured (dark grey), sequence conserved (teal), and are potentially functional (blue). Bottom: RNAcofold models between 9530-9570/9820-9890 with the potential miR19a-3p binding site. **b**, Bar charts showing the levels of miR-19a-3p and control RNU48 at 0, 2, 16, and 24 hours post-ZIKV infection using qPCR analysis. **c**, Bar chart showing the levels of PTEN 2 hours post ZIKV infection by qPCR analysis. **d**, Boxplot showing distribution in gene expression changes of miR-19a-3p targets and non-targets 2 hours post-ZIKV infection in Huh7 cells.

Fig S4

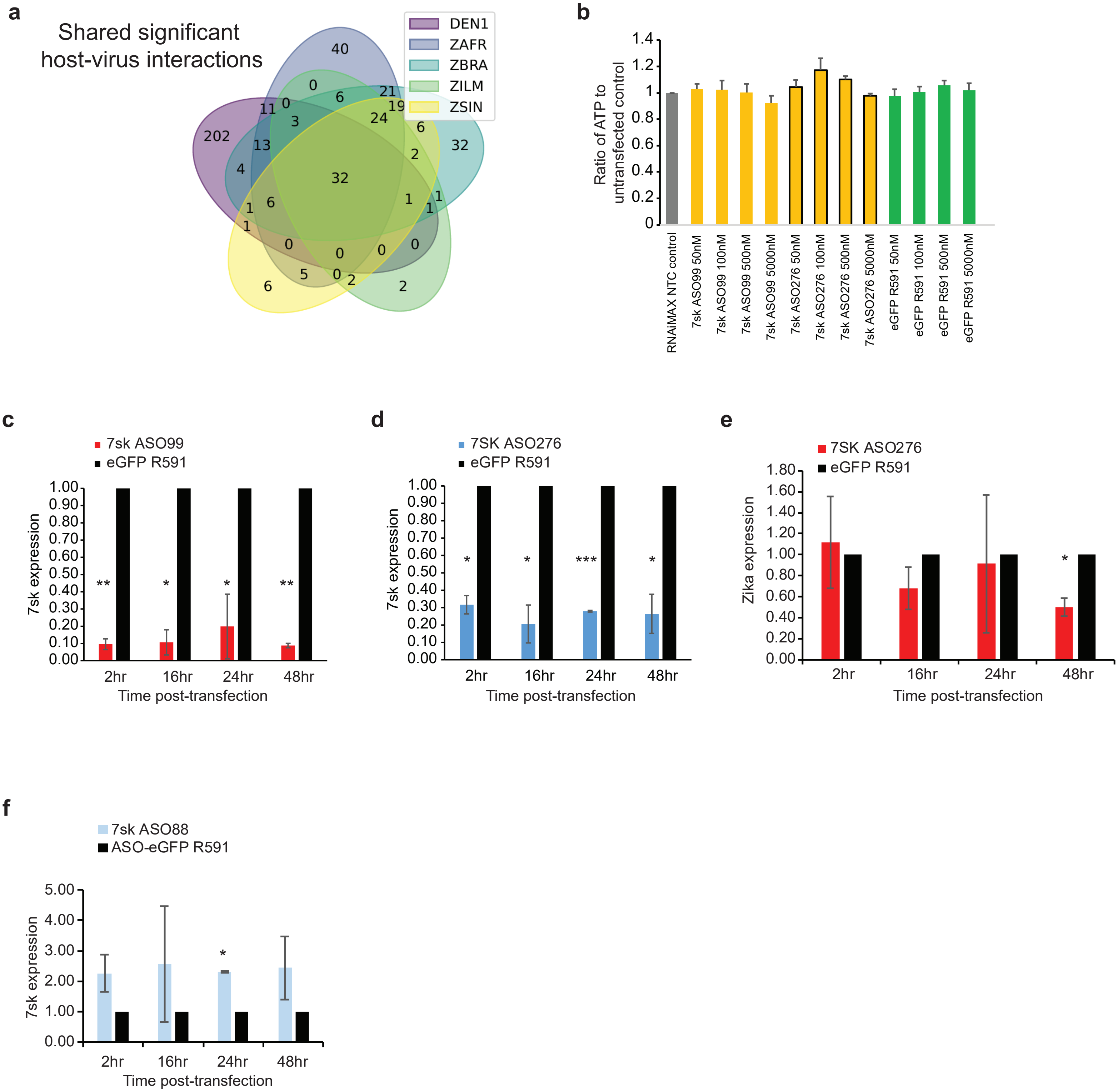
a
Shared significant
host-virus interactions
DEN1
ZAFR
ZBRA
ZILM
ZSIN
b
Ratio of ATP to
untransfected control
RNAiMAX NTC control
7sk ASO99 50nM
7sk ASO99 100nM
7sk ASO99 500nM
7sk ASO99 5000nM
7sk ASO276 50nM
7sk ASO276 100nM
7sk ASO276 500nM
7sk ASO276 5000nM
eGFP R591 50nM
eGFP R591 100nM
eGFP R591 500nM
eGFP R591 5000nM
c
7sk ASO99
eGFP R591
7sk expression
2hr
16hr
24hr
48hr
Time post-transfection
d
7SK ASO276
eGFP R591
7sk expression
2hr
16hr
24hr
48hr
Time post-transfection
e
7SK ASO276
eGFP R591
Zika expression
2hr
16hr
24hr
48hr
Time post-transfection
f
7sk ASO88
ASO-eGFP R591
7sk expression
2hr
16hr
24hr
48hr
Time post-transfection

Figure S4. ZIKV:non-coding RNA interaction statistics. a, Venn diagram showing the overlap in significant interactions between the different viruses. 32 host RNAs bind to all 5 viruses. **b**, Bar charts showing the average ratio of ATP in cells transfected with different concentrations of ASOs versus ATP in cells that are untransfected. **c,d**, Bar chart showing the levels of 7SK in Huh7 cells 2, 16, 24, 48 hours after transfection of two independent 7SK ASOs, ASO99 (**c**) and ASO276 (**d**) to knock it down. **e**, Bar chart showing the amount of ZIKV present in Huh7 cells 2, 16, 24, 48 hours after 7SK knockdown using ASO276, by qPCR analysis. ZIKV levels are decreased when 7SK levels are decreased. **f**, Bar chart showing the levels of 7SK in Huh7 cells 2, 16, 24, and 48 hours after transfection of 2'O-methylated ASO oligo to 7SK. Transfecting 2'O-methylated ASO against 7SK does not knock down 7SK inside Huh7 cells. **c-f**, *, **, *** indicate p-values <= 0.05, 0.01, and 0.001 respectively, using Student's T-test.

Fig S5

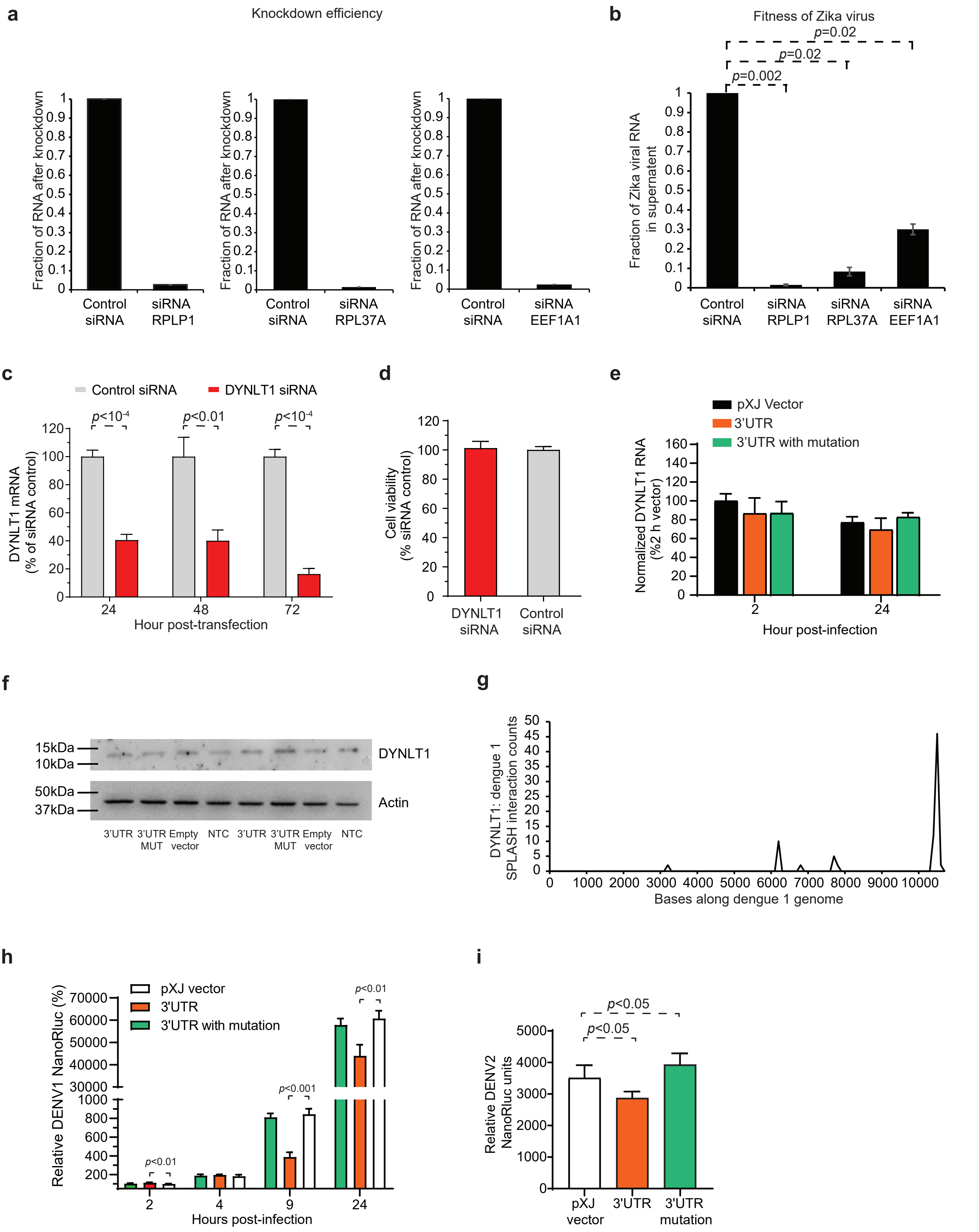
a
Knockdown efficiency
Fraction of RNA after knockdown
1
0.9
0.8
0.7
0.6
0.5
0.4
0.3
0.2
0.1
0
Control siRNA
siRNA RPLP1
siRNA RPL37A
siRNA EEF1A1
b
Fitness of Zika virus
p=0.02
p=0.02
p=0.002
Fraction of Zika viral RNA in supernatent
Control siRNA
siRNA RPLP1
siRNA RPL37A
siRNA EEF1A1
c
Control siRNA
DYNLT1 siRNA
p<10-4
p<0.01
p<10-4
DYNLT1 mRNA (% of siRNA control)
120
100
80
60
40
20
0
24
48
72
Hour post-transfection
d
Cell viability (% siRNA control)
DYNLT1 siRNA
Control siRNA
e
pXJ Vector
3'UTR
3'UTR with mutation
Normalized DYNLT1 RNA (%2 h vector)
160
140
2
24
Hour post-infection
f
15kDa
10kDa
50kDa
37kDa
DYNLT1
Actin
3'UTR
3'UTR MUT
Empty vector
NTC
g
DYNLT1: dengue 1 SPLASH interaction counts
50
45
35
30
25
15
10
5
1000
2000
3000
4000
5000
6000
7000
8000
9000
10000
Bases along dengue 1 genome
h
pXJ vector
3'UTR
3'UTR with mutation
Relative DENV1 NanoRluc (%)
70000
60000
50000
40000
30000
1000
800
600
400
200
p<0.01
p<0.001
p<0.01
2
4
9
24
Hours post-infection
i
p<0.05
p<0.05
Relative DENV2 NanoRluc units
5000
4000
3000
2000
1000
pXJ vector
3'UTR
3'UTR mutation

Figure S5. DYNLT1 inhibits DENV and ZIKV replication. a, Bar plots showing the knockdown efficiencies of siRNAs against RPLP1, RPL37A, and EEF1A1. All 3 transcripts are knocked down to >90% of their original RNA amount. RPLP1 is a positive control known to affect ZIKV virus replication. RPL37A and EEF1A1 RNAs are identified as interactors of ZIKV RNA. **b**, Bar plots showing the amount of ZIKV virus RNA detected in the supernatant 24 hours post-ZIKV infection, after knockdown of the respective genes. Knockdown of RPL37A and EEF1A1 significantly reduced the amount of virus production from the cells after 24 hours. **c,** Knockdown of DYNLT1 expression after siRNA transfection. Huh7 cells were transfected with 20 nM of DYNLT1 siRNA or a control siRNA. At 24, 48, and 72 h post-transfection, the intracellular DYNLT1 mRNAs were quantified by RT-qPCR. The relative mRNA levels were obtained by normalizing the DYNLT1 mRNAs of each group to those of the siRNA control group. Means and SDs from four independent experiments are presented. **d**, Knockdown of DYNLT1 siRNA does not affect cell viability. Huh7 cells were transfected with 20 nM of DYNLT1 siRNA or a control siRNA. At 48 h post-transfection, cell viability was measured using the CellTiter-Glo® Luminescent cell viability assay. Data were normalized to the control groups. Means and SDs from three independent experiments are presented. **e**, Overexpression of DYNLT1 3'UTR marginally affects endogenous DYNLT1 mRNA. Huh7 cells were transfected with 200 ng of DYNLT 3'UTR, DYNLT1 3'UTR with mutation, or pXJ vector. At 24 h post-transfection, cells were infected with recombinant ZIKV strain PRVABC59 containing a nanoluciferase gene at an MOI of 0.5. At 2 h and 24 h post-infection, the intracellular DYNLT1 mRNAs were quantified by RT-qPCR. The relative mRNA levels were obtained by normalizing the DYNLT1 mRNAs of each group to those of the control group at 2 h post-infection. Means and SDs from four independent experiments are presented. **f**, Western blot showing the protein levels of DYNLT1 in Huh7 cells after overexpression of DYNLT1 3'UTR (lanes 1 and 5), DYNLT1 mutant 3'UTR (lanes 2 and 6), and empty vector (lanes 3 and 7). **g**, Line plot showing the locations along DENV-1 that binds to DYNLT1. The Y-axis indicates the number of interaction counts between DENV-1 and DYNLT1 for that location. **h**, DYNLT1 3'UTR inhibits DENV-1 replication. Huh7 cells were transfected with 400 ng of DYNLT 3'UTR, DYNLT1 3'UTR with mutation, or pXJ vector. At 24 h post-transfection, cells were infected with recombinant DENV-1 strain West Pac containing a nanoluciferase gene at an MOI of 1.0. At given time points, cells were harvested. The relative luciferase signals were obtained by normalizing the luciferase readouts of each group to those of control at 2 h post-infection. Means and SDs from four independent experiments are presented. **i**, DYNLT1 3'UTR suppresses DENV-2 replication. Huh7 cells were transfected with 200 ng of DYNLT 3'UTR, DYNLT1 3'UTR with mutation, or pXJ vector. At 24 h post-transfection, cells were infected with recombinant DENV-2 D2Y98P containing a nanoluciferase gene at an MOI of

1.0. At given time points, cells were harvested. The luciferase signals were measured. Means and SDs from four independent experiments are presented.
