# Supplementary material for: Dengue and Zika RNA-RNA interactomes reveal pro- and anti-viral RNA in human cells
Source: Genome Biol. 2023 Dec 5;24:279. doi: 10.1186/s13059-023-03110-9 (PMC10696742; doi:10.1186/s13059-023-03110-9)
Supplement: Supplementary file 7 — Additional file 7. Review history. [file 13059_2023_3110_MOESM7_ESM.docx]

Review History

**First round of review**

**Reviewer 1**

**Are you able to assess all statistics in the manuscript, including the appropriateness of statistical tests used?** Yes, and I have assessed the statistics in my report.

**Comments to author:**

The article is well-written and the results are convincing. The methods are appropriate, as well as the statistical analyses. The code is not available yet but I am confident it will be upon acceptance of the manuscript.

I only have one request for the authors. It would be interesting to better elucidate the mechanism by which miR-19a-3p acts as a pro-viral factor. In a previous report, Ziv and colleagues (https://pubmed.ncbi.nlm.nih.gov/30202058/) reported similar findings for miR-21, showing that reducing miR-21 levels also reduced ZIKV RNA levels.

In a recent review, Spitale and Incarnato (https://www.nature.com/articles/s41576-022-00546-w) suggested that interaction with miR-21 might drive the switch between genome cyclization and genome translation. In this regard, it would be interesting to understand whether the interaction with miR-19a-3p might be acting through a similar mechanism. Are there any long-range interactions that are mutually-exclusive with the miR-19a-3p binding site on the ZIKV genome? I would suggest the authors to perform ribosome profiling on the virus in miR-19a-3p mimic and antagomiR cells as compared to control cells to see if they do observe any change in ribosome occupancy that would support this mechanism.

**Reviewer 2**

**Are you able to assess all statistics in the manuscript, including the appropriateness of statistical tests used?** Yes, and I have assessed the statistics in my report.

**Comments to author:**

Liao and co-authors revealed the RNA-RNA interactomes of both Dengue and Zika, where they discovered the host interacted RNAs, including ncRNAs and mRNAs. The authors further validated the functions of these interacted host RNAs in regulating viral activities. The work is comprehensive, and the results provide new insights into viral-host interactions.

My main comments are as follows.

Is there any preference for the sequence motifs across these RNA-RNA interaction sites?

The authors have profiled several Zika strains (African, Brazil, ILM and Singapore). Are there any variations of RNA-RNA interactions on the common host RNAs among these strains?

It would be interesting if the authors could evaluate the conservation of the RNA-RNA interaction sites on the host RNAs.

Identifying 3'UTR of DYNLT1 as the Zikv interacted RNA is exciting. Do any RNA binding proteins bind to these interaction sites?

**Authors Response**

We thank the reviewer for his/her comments, which have significantly improved the manuscript. Below are our point-by-point comments.

Reviewer reports:

Reviewer #1: The article is well-written and the results are convincing. The methods are appropriate, as well as the statistical analyses. The code is not available yet but I am confident it will be upon acceptance of the manuscript.

We thank the reviewer for his/her positive comments.

I only have one request for the authors. It would be interesting to better elucidate the mechanism by which miR-19a-3p acts as a pro-viral factor. In a previous report, Ziv and colleagues ([https://urldefense.com/v3/__https://pubmed.ncbi.nlm.nih.gov/30202058/__;!!NLFGqXoFfo8MMQ!oTAymYFBluKir47yvvBWP9oOZlSrPOaZ_DrNIM0VbCrIIYXlQmzDrOhNvns1mNLuXgLnoj6nYJWdxAPbFMcIksNCMA$](https://urldefense.com/v3/__https:/pubmed.ncbi.nlm.nih.gov/30202058/__;!!NLFGqXoFfo8MMQ!oTAymYFBluKir47yvvBWP9oOZlSrPOaZ_DrNIM0VbCrIIYXlQmzDrOhNvns1mNLuXgLnoj6nYJWdxAPbFMcIksNCMA$) ) reported similar findings for miR-21, showing that reducing miR-21 levels also reduced ZIKV RNA levels.
In a recent review, Spitale and Incarnato (<https://www.nature.com/articles/s41576-022-00546-w>) suggested that interaction with miR-21 might drive the switch between genome cyclization and genome translation. In this regard, it would be interesting to understand whether the interaction with miR-19a-3p might be acting through a similar mechanism. Are there any long-range interactions that are mutually-exclusive with the miR-19a-3p binding site on the ZIKV genome?

We thank the reviewer for his/her comments. We have now examined all the pair-wise interactions within the Zika genome that could potentially compete with the miR-19 binding site. We identified 2 main areas of interaction that can potentially compete with miR-19a-3p interaction at the positions 9680-9700 (Figure 1). The first region forms a strong local stem loop in the absence of miR-19a-3p, so miR-19a-3p potentially disrupts this stem upon binding.

Figure 1. Schematic of potential RNA structures present on Zika before miR-19a-3p binding. Top left: Top: Arc plots showing the global pair-wise RNA-RNA interactions along the Zika ILM genome. The heat map shows the bases along the Zika genomes that can pair with the miR-19a-3p binding site. The red arcs below indicate the local pairings in this region that is supported by SPLASH. Top right: Base-pairing along the Zika genome before and after binding to miR-19a-3p. Bottom: Two regions can pair with the miR-19a-3p binding site. We are showing the schematic of how the pairing could look like before miR-19a-3p binding. The miR-19a-3p site is in red.

In the second region, bases 9820-9890, which is 150 bases downstream (9820-9890) of the miR-19a-3p binding site, pairs with the miR-19a-3p binding site at 9684. miR-19a-3p binding hence potentially disrupts this 150 base-pair interaction and releases the 9820-9890 region for other interactions. Interestingly, this 9820-9890 region falls within a highly structurally conserved region that we previously identified across different Zika strains (green arrow, Figure 2). As such, we hypothesize that binding of miR-19a-3p to the Zika genome can potentially release this region to enable it to fold into its proper structure that is important for viral function. We have now included this as Supp. Figure 3a in our manuscript.

*Figure 2. The second potential interaction site on Zika genome (green arrow) falls in structured region that is structurally conserved across the different Zika genomes.*

I would suggest the authors to perform ribosome profiling on the virus in miR-19a-3p mimic and antagomiR cells as compared to control cells to see if they do observe any change in ribosome occupancy that would support this mechanism.

As described above, there would be many reasons why miR-19a-3p promotes Zika growth, including both direct (above) and indirect strategies by changing the gene expression of other miR-19a-3p target genes in the cell (Supp. Figure 3b,c). However, following the reviewer’s suggestions, we have performed ribosome profiling in the absence and presence of the miR-19a-3p mimic. Unfortunately we did not see a significant difference in translation efficiency (defined as the number of footprints/mRNA count) of Zika genome in the absence or presence of miR-19a-3p, suggesting that miR-19a-3p is not likely to be acting at the level of translation for Zika (Figure 3, below).

Figure 3. Top: Normalized ribosome footprints along the Zika genome in Huh7 cells that have been transfected with either miR-19a-3p mimic control (blue) or miR-19a-3p mimic (orange). Bottom: Left: Barplots showing the translation efficiency (TE) along the Zika genome (calculated as the sum of footprints/sum of total mRNA reads along Zika) for 2 biological replicates. Right, Barplots showing the number of total reads mapped to ZIKV genome in control versus mimic transfected cells. We did not see a significant change in TE for Zika in the presence of miR-19a-3p, despite seeing an expected increase in the number of reads on ZIKV in the presence of mimic.

Reviewer #2: Liao and co-authors revealed the RNA-RNA interactomes of both Dengue and Zika, where they discovered the host interacted RNAs, including ncRNAs and mRNAs. The authors further validated the functions of these interacted host RNAs in regulating viral activities. The work is comprehensive, and the results provide new insights into viral-host interactions.

We thank the reviewer for his/her positive comments.

My main comments are as follows.
Is there any preference for the sequence motifs across these RNA-RNA interaction sites?

We performed enrichment analysis for the sequence motifs across these RNA-RNA interaction sites- however unfortunately we could not identify a common sequence motif. This is probably because the viruses use different sequences to interact with different host sequences along its genome.

The authors have profiled several Zika strains (African, Brazil, ILM and Singapore). Are there any variations of RNA-RNA interactions on the common host RNAs among these strains?

We thank the reviewer for his/her insightful comments. We have now compared all host-virus RNA-RNA interactions across the four Zika strains for the shared overrepresented SPLASH interactors (140 interactions in total) and we observed that the RNA-RNA interactions between the different Zika viruses are highly similar. The following figure shows the Pearson correlation of the host interaction pattern for GADPH as an example (Figure 4).

Figure 4. Locations of RNA-RNA interactions with GAPDH along ZIKV/DENV genomes. The viruses largely use the same locations to interact with GAPDH, although the Zika viruses are much more similar to each other than to Dengue.

Globally, across the 140 over-represented virus-host interactions, we notice that a significant proportion of interactions are well conserved across the different Zika viruses (exceeding a correlation of 0.5 across the full genome), indicating that these interactions are robust across the different Zika viruses (Figure 5).

Figure 5. Histogram showing the distributions of correlations across different ZIKV viruses for their interactions with host-RNAs. We see a generally high correlation indicating that most of the ZIKV use the same regions (and similar intensities) to interact with host RNAs.

It would be interesting if the authors could evaluate the conservation of the RNA-RNA interaction sites on the host RNAs.

We thank the reviewer for his/her insightful comments. We have also examined the interactions on the host side and find them to be equally well conserved, with a large number of regions in host that bind to all of the four Zika viruses (Figure 6).

Figure 6. Histogram showing the distributions of correlations on host RNAs for their interactions with different Zika viruses. We see a generally high correlation indicating that when a host RNA interact with one ZIKV using a particular site, it also interacts with other ZIKV using the same site and intensity.

We also show specific examples of interaction sites on GAPDH that interact with the different Zika and dengue viruses (Figure 7).

*Figure 7. Line plots showing the location of interaction between GAPDH and ZIKV along the GAPDH mRNA. Right: heatmap showing a high correlation between GAPDH and different ZIKV genomes, indicating that when GADPH uses one region to interact with one ZIKV, the same region is also highly likely to be used to interact with other ZIKV.*

To further investigate whether the host-virus interactions occur in conserved regions along the host genome, we extracted the PhastCons 100 vertebrae conservation track from USCS genome browser and compared evolutionary conservation of host interacting locations (>5 SPLASH reads) with non-interacting locations. We observed a significant shift towards more conserved regions for host-virus interaction than for non-virus interacting regions, indicating that the viruses indeed bind to more conserved regions (Figure 8). We have now added this as our Supp. Figure 2d.

Identifying 3'UTR of DYNLT1 as the Zikv interacted RNA is exciting. Do any RNA binding proteins bind to these interaction sites?

Figure 8. Histogram showing the distribution of PhastCons score for regions along host RNAs that interact with ZIKV genomes (red), and that do not interact with ZIKV genomes (black).

We thank the reviewer for his/her insightful comments. We investigated the interactions of RNA-binding proteins with the DYNLT1 binding site on the Zika genome using sequence motifs. Out of 159 unique RBPs for which we could find a total of 116 annotated interaction motifs, we matched these motifs to the ZIKV-ILM genomic RNA using a position score matrix and a threshold of 50% of maximum probability for a match. We identified a total of 144 RBP motifs in the region of 10660-10680, corresponding to 50 RBPs. To identify a more confident set of RBPs, we intersected these 50 RBPs against an experimental ZIKA protein interactome dataset whereby the authors pulled down the Zika genome and performed mass spectrometry analysis to identify interacting proteins. Filtering for real Zika interactors, we identified a total of 24 RBPs that could bind to ZIKV at the DYNLT1 site and potentially compete with DYNLT1 for interaction. We have now included these RBPs as an additional Supp. Table 5 in our manuscript.

**Second round of review**

**Reviewer 1**

I am content with the authors' response.

**Reviewer 2**

The authors have fully addressed my comments. There is no further comments
